# The changes in dioxin and PCB concentrations in different feed materials over time – preliminary study

**DOI:** 10.2478/jvetres-2025-0065

**Published:** 2025-11-11

**Authors:** Marek Pajurek, Małgorzata Warenik-Bany, Szczepan Mikołajczyk, Wojciech Jerzy Pietroń

**Affiliations:** Department of Chemical Research of Food and Feed, National Veterinary Research Institute, 24-100 Puławy, Poland

**Keywords:** congeners, dioxins, feeds, PCBs, time trends

## Abstract

**Introduction:**

Polychlorinated dibenzo-p-dioxins and dibenzofurans (PCDD/PCDFs) and polychlorinated biphenyls (PCBs) are a group of undesirable chemical substances classified as persistent organic pollutants (POPs). They enter the bodies of humans and animals mainly through the digestive tract. Therefore, the safety of the food chain with specific regard to keeping out these POPs depends heavily upon their elimination from animal feed. The aim of this study was to determine trends in PCDD/PCDF/PCB concentrations in three feed matrices over a six-year period.

**Material and Methods:**

Altogether, 360 feed samples were analysed using the isotope-dilution technique with high resolution gas chromatography coupled with high resolution mass spectrometry. Analysis of the variability of PCDD/PCDF, dioxin-like-PCB and non-dioxin-like-PCB concentrations by Mann–Kendall test and regression analysis for mean and median values was performed for the three most common feed categories (plant materials, fishmeal and feed mixtures).

**Results:**

For two of the three types of feed materials analysed, changes in trends in the concentrations of the tested compounds over time were observed.

**Conclusion:**

Considering the very limited amount of feed material analysis data on which to assess trends in dioxin concentrations, it is necessary to continue research on a wider group of feed types.

## Introduction

Dioxins are organochlorine compounds that are produced in combustion processes and are by-products of many chemical processes. This group includes polychlorinated dibenzo-p-dioxins (PCDDs), polychlorinated dibenzofurans (PCDFs) and polychlorinated biphenyls (PCBs). These compounds are undesirable chemicals, classified as persistent organic pollutants, and characterised by high persistence in the environment and strong toxicity. The strong affinity of dioxins for fats causes their bioaccumulation and deposition in the tissues of living organisms (4). Dioxins may cause chronic toxic effects when exposure times are long (3). The main route of human exposure to these compounds is the diet, through which approximately 80% of exposure takes place, with the dominant source being food of animal origin. Therefore, the feeds provided to animals contribute significantly to the presence of these compounds in food (13, 15, 16). Even small amounts of dioxins and PCBs in animal feed can cause unacceptable levels of these contaminants to accumulate in animal tissue and persist in animal products (14).

Feed with an appropriate composition is provided to domestic animals to ensure optimal production of milk, meat and eggs. Therefore, it is very important that feed is not a source of contamination that may negatively affect animal health and the safety of food of animal origin. Many scientific reports indicate that for farm animals, the main route of exposure to dioxins and PCBs is feed (9, 11–13, 16). Dioxins can enter the raw materials of feed through various routes; emission sources can be divided into industrial and non-industrial. Dioxins and furans have never been produced intentionally, but have been generated as by-products of certain industrial processes (*e.g*. metallurgical processes, paper bleaching and pesticide production), which subsequently pollute terrestrial and aquatic environments (5, 10). Non-industrial sources can include volcanic eruptions and forest fires. At present, combustion processes (*e.g*. burning coal in stoves, domestic waste incineration and internal combustion engines) are the most constant polluters of the local environment. Faulty production and inappropriate processing and transport of feed and feed materials can also lead to their contamination with dioxins and PCBs (13). Deliberate illegal action may also cause contamination of feed, as was the case in the Belgian PCB/dioxin crisis (2). Given all these possible routes of contamination of feed materials, the primary control measure is the most practical one: to limit the occurrence of contamination in the production and processing of feed materials. This can be achieved through constant laboratory quality control that will eliminate the current dioxin and PCB threat and identify new sources of it. This is the basic measure that currently assures food safety, reducing the food consumer’s exposure to dioxins and PCBs. This article is the outcome of the conduct of extensive multiyear studies, and collates and presents data on the trends in changes of dioxin and PCB concentrations in feeds available on the domestic market, including feed materials.

## Material and Methods

### Sampling and sample collection

The study material consisted of samples of three types of feed (feed materials of plant origin, compound feeds and fishmeal) taken as part of the Polish national official monitoring programme. The pool of 360 samples also included imported feed materials collected by Border Veterinary Inspectorates and others sent by producers and exporters. The numbers of samples tested by category according to Regulation 277/2012/EU and by year covering the study period (2013–2018) are presented in Table 1. The representativeness of the sampling was ensured by the application of the current requirements of the EU regulations by the veterinary inspection staff taking the samples.

**Table 1.** Feed categories (in the Regulation 277/2012/EU scheme) and number of samples tested per year

Research material	2013	2014	2015	2016	2017	2018	2013–2018
materials of plant origin^1^	7	40	45	37	34	20	183
fishmeal^2^	22	9	21	18	11	16	97
compound feeds^3^	11	2	12	14	18	23	80
Sum	40	51	78	69	63	59	360

1 – cereal grains (wheat, rye, maize and oats), dried beet pulp and dried plants;

2 – Baltic fishmeal;

3 – complete and complementary feeds

### Analytes of interest

The analytes of interest were seven PCDD congeners, ten PCDF congeners, twelve dioxin-like PCB (dl-PCB) congeners (PCB 77, 81, 105, 114, 118, 123, 126, 156, 157, 167, 169 and 189) and six non-dioxin-like PCB (ndl-PCB) congeners (PCB 28, 52, 101, 138, 153 and 180).

### Reagents and chemicals

All of the organic solvents were of suitable purity for the residue analysis. Normal hexane, dichloromethane, toluene and n-nonane were supplied by LGC Standards (Wesel, Germany). Sodium sulphate and 98% ACS-grade sulphuric acid were purchased from Merck (Darmstadt, Germany), and diatomaceous earth from Restek (Bellefonte, PA, USA). Helium (purity 99.9999%) and nitrogen (99.999%) were sourced from Messer (Gumpoldskirchen, Austria). All standards were purchased from the Cambridge Isotope Laboratory (Andover, MA, USA) and Wellington Laboratories Inc. (Guelph, ON, Canada).

### Sample preparation

After initial sample preparation and grinding, the corresponding sample weight (10 g–20 g) was extracted using an ASE 300 accelerated solvent extractor (Dionex, Sunnyvale, CA, USA). One of several extraction programmes and solvent mixtures was used each time as suited the type of sample under analysis. Purification of samples was performed using multistage column chromatography with modified silica gel, Florisil and Carbopack C. Standards of recovery (^13^C12 PCB 111 and ^13^C12 1,2,3,4-tetrachlorodibenzo-p-dioxin (TCDD)) were added to concentrated eluates of each fraction (PCDD/PCDFs, non-ortho dl-PCBs and mono-ortho dl-PCBs).

### Instrumental analysis

The concentrated eluates of the individual fractions (PCDD/PCDFs, non-ortho dl-PCBs and mono-ortho PCBs) were instrumentally analysed. The technique was combined high-resolution gas chromatography/high-resolution mass spectrometry in an Ultra Trace GC gas chromatograph (Thermo Fisher Scientific, Milan, Italy) with an AS2000 (CTC Analytics/Thermo Fisher Scientific, Zwingen, Switzerland) automatic sample feeder coupled to a MAT95XP (Thermo Fisher Scientific, Bremen, Germany) high resolution mass spectrometer with dual focus (magnetic and electrical) and inverted Nier–Johnson geometry. The mass spectrometer was operated in electron ionisation mode under conditions that provided a resolution in excess of 10,000 for the full range of mass spectra collected. A 60 m × 0.25 mm DB-5 ms capillary column with a 0.1 μm weakly polar stationary phase was used.

### Quality assurance and quality control

Research quality control (internal and external) included measurement of recoveries of internal standards labelled with the ^13^C carbon isotope (1,2,3,4-TCDD and PCB-111), analysis of reagent blank samples, the use of certified reference materials (BCR-607 milk powder) and participation in proficiency tests organised by the European Union Reference Laboratory for Persistent Organic Pollutants (POPs) in Feed and Food (Freiburg, Germany).

### Statistical analysis

Mean and median values for each annual period were calculated and slope coefficients were determined using linear regression. The fit of the function to the measured data was verified with the value of the R^2^ determination coefficient, applying the generally accepted criterion of 0.6 ≤ R^2^ ≤1 for satisfactory or very good fit. In addition, the non-parametric Mann–Kendall (M-K) test was used to assess time trends in concentrations, where a minimum of five time intervals was established as necessary for reliable trend detection at the P-value = 0.05 significance level.

## Results

For the three feed categories, namely plant feed materials, fish meal and compound feeds (for livestock), the variability of PCDD/PCDF, dl-PCB and ndl-PCB concentrations was analysed over a defined time interval, which covered the six-year period 2013–2018.

### Plant feed materials

A total of 183 samples of plant feed materials were analysed. The average concentrations of PCDD/PCDFs in this material were close to 34% of the maximum limit (ML), and those of PCDD/PCDF/dl-PCBs combined constituted approximately 23% of the ML. There were only trace levels of ndl-PCBs in the plant material, and their concentrations amounted to around 1% of the ML.

**Fig. 1.**
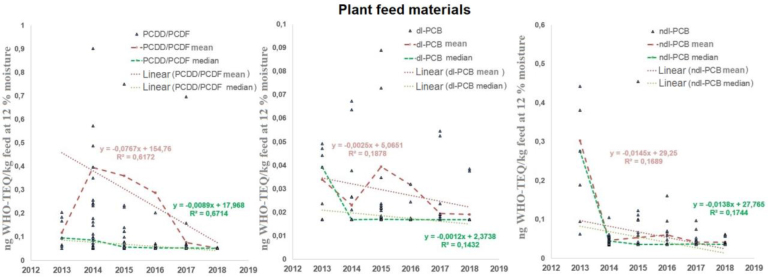
Changes in the World Health Organization toxicity equivalent (WHO-TEQ) concentrations of polychlorinated dibenzo-p-dioxins and dibenzofurans (PCDD/PCDFs), dioxin-like polychlorinated biphenyls (dl-PCBs) and non-dioxin-like PCBs (ndl-PCBs) over time for plant feed materials

For this category of feed with respect to PCDD/PCDFs, the results of the M-K test for the median and the regression analyses for the median and mean showed a decreasing trend (Fig. 1). The determination coefficients met the satisfactory criterion for 67% of regression analyses using medians and 62% of those using means. In the case of dl-PCBs, the approximation curves for the median and mean indicated a negative trend, but the quality of fit of the curves in both cases was unsatisfactory (R2 < 0.19), while the M-K test indicated that there was a decreasing trend only for the median. Thus, it cannot be clearly concluded that there was a decreasing trend for dl-PCBs. For ndl-PCBs, neither the M-K test nor the determination coefficients showed a trend. The coefficient in both cases was calculated with unsatisfactory values (R2 < 0.17). However, it can be noted that the slope coefficients of the regression curves for the median and mean had negative values, so if there had been a trend in changes for ndl-PCBs, it would also have been a decreasing trend.

### Compound feeds (for livestock)

**Fig. 2.**
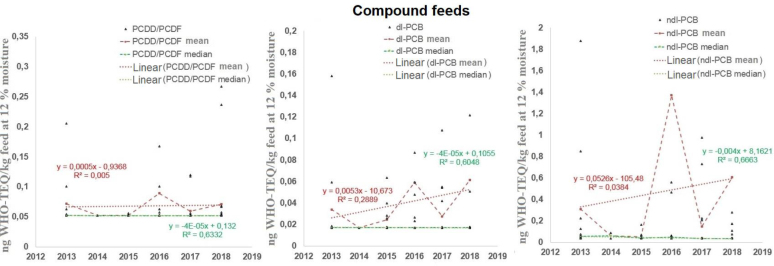
Changes in the World Health Organization toxicity equivalent (WHO-TEQ) concentrations of polychlorinated dibenzo-p-dioxins and dibenzofurans (PCDD/PCDFs), dioxin-like polychlorinated biphenyls (dl-PCBs) and non-dioxin-like PCBs (ndl-PCBs) over time for compound feeds

The M-K test and the analysis of regression values for the mean values of feed mixtures for farm animals in terms of PCDD/PCDF concentrations indicated no trend, and the slope coefficient of the curve for mean values was positive (Fig. 2). However, for the medians, the approximation curve indicated the occurrence of a very slow negative trend, supported by a satisfactory regression curve fit (R^2^ = 0.63). A similar situation occurred for dl-PCBs: the M-K test and the unsatisfactory (R^2^ < 0.29) fit of the regression curve for the mean values indicated the absence of a specific trend. However, the approximation curve for the median values in individual years indicated a very slow negative trend, and here, determination coefficients indicated satisfactory fit for 60% of the analyses. In the case of ndl-PCBs, both the M-K test and the low value of the determination coefficients of mean value regressions showed there to be no trend. However, the median values for the M-K test and the regression analysis’ satisfactory result (R^2^ = 0.67) indicated a decreasing trend. These values for all analysed compounds support the conclusion that there was a very slow decreasing trend in changes in feed mixtures.

### Fishmeal

In this category of feed materials, a total of 97 samples were tested. The average concentrations of PCDD/PCDFs were around 42% of the ML, and those of PCDD/PCDF/dl-PCBs were 29% of the ML. In the case of ndl-PCBs, the average levels were approximately 20% of the ML.

**Fig. 3.**
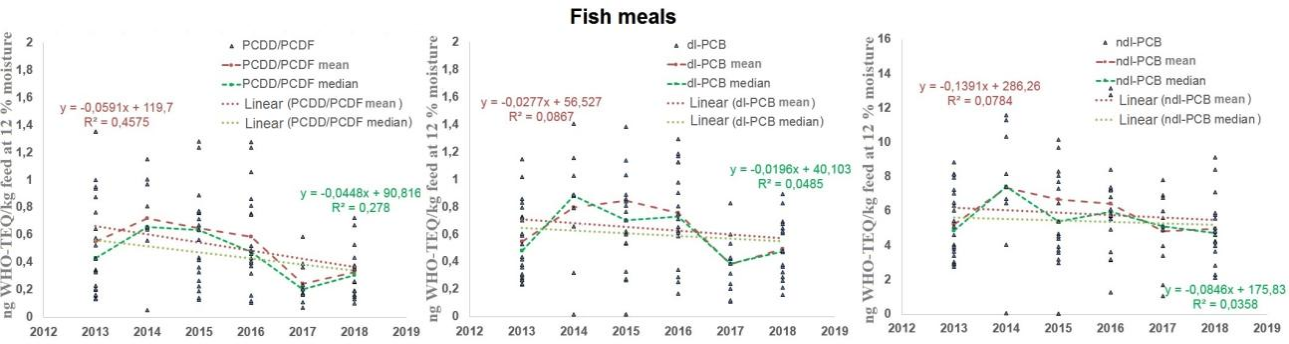
Changes in the World Health Organization toxicity equivalent (WHO-TEQ) concentrations of polychlorinated dibenzo-p-dioxins and dibenzofurans (PCDD/PCDFs), dioxin-like polychlorinated biphenyls (dl-PCBs) and non-dioxin-like PCBs (ndl-PCBs) over time for fishmeal

Mann–Kendall tests performed for both the median and mean values for PCDD/PCDFs, dl-PCBs and ndl-PCBs in the analysed annual periods showed no trends of change. Neither did regression analysis of the medians and means, as evidenced by the low value of the determination coefficients (Fig. 3). However, when analysing the slope coefficients of the curves for both the medians and the means for all analysed groups, they emerge as having negative values. Therefore, it can be seen that if there had been a trend to the changes, it would have been a decreasing one. However, further observations of dioxin and PCB concentrations should be carried out in the following years to confirm the trend.

## Discussion

Animal products are everyday components of the diet for many consumers. The safety of animal-origin foods is inextricably linked to the quality of the feed given to animals, directly or indirectly. Without using safe and good-quality feed, the legal and production requirements in the breeding and raising of farm animals cannot be fulfilled, and the safety and quality of the product reaching the consumer may be compromised. Continuous reduction of consumer exposure to dioxins and PCBs is a goal of EU strategy, and our research was aligned with this strategy and complemented the continuous monitoring of feed and feed materials for the presence of these contaminants.

The mean PCDD/PCDF concentrations in the feed materials analysed in the study were in the following order: fishmeal > plant-origin feed materials > compound feeds. For compound feeds, the average dioxin concentrations were at low levels close to the limit of quantification for the analytical method; however, even lower levels for this feed category were presented in the 2018 European Food Safety Authority (EFSA) report (8). Concentrations of PCDD/PCDFs in fishmeal reported by Adamse *et al*. (8) and the EFSA showed levels almost twice as low as the results in this study. For feed materials of plant origin, the results obtained were similar to the level reported by Adamse *et al*. (1) in 2015, while the EFSA report predating this and EFSA data published after it showed lower results in these materials (6, 8). The highest average concentrations of dl-PCBs in the conducted studies were found in fishmeal. In contrast, feed mixtures and plant feed materials contained comparable trace amounts of these compounds. The highest average concentrations of ndl-PCBs were also determined in fishmeal, and the next-highest in feed mixtures. In plant feed materials, ndl-PCBs were determined at a level below 1% of the permissible limit.

The main objective of this work was to analyse the variability of PCDD/PCDF, dl-PCB and ndl-PCB concentrations by carrying out an M-K test and regression analysis for the mean and median values over a time period from 2013 to 2018 for three feed categories. For feed materials of plant origin, of all the analysed compound groups, a decreasing trend in changes was only shown for PCDD/PCDFs. For compound feeds, in contrast, a slow decreasing trend was found for all analysed groups of dioxin, dl-PCB and ndl-PCB compounds based on regression analysis for the median values. The third group of feed materials we analysed was fishmeal, and in this case no trends in changes of concentrations of these compounds over time could be found.

It is worth noting that the EFSA did not analyse time trends in its publications from 2010, 2012 or 2018 (6–8). Test results for PCDD/PCDFs and dl-PCBs in the same three categories of feed from the Netherlands in the period of 2003–2010 did not show any trend, and the concentrations of the tested compounds were at the same level as ours in the analysed period, which overlaps with the period of our sampling (1, 17).

## Conclusion

Given the limited availability of data on trends of changes in analyses of dioxins and PCBs in feed materials, there is a clear need for continued research in this area. Expanding the scope to include a broader range of feed types and ingredients is essential for a more comprehensive assessment of both animal and consumer exposure to these persistent environmental contaminants.
